# Safety and efficacy of DEB-TACE in combination with lenvatinib and camrelizumab for the treatment of unresectable hepatocellular carcinoma (uHCC): a two-centre retrospective study

**DOI:** 10.3389/fimmu.2024.1422784

**Published:** 2024-10-22

**Authors:** Zhang Xuexian, Wang Ruidong, Ding Yuhan, Li Qingwei, Xiong Feng, Ren Hong, Zhang Jun, Li Wei

**Affiliations:** ^1^ Department of Vascular Intervention, Jingmen People’s Hospital, Jingmen, Hubei, China; ^2^ Interventional Department, Qujing Second People’s Hospital, Qujing, Yunnan, China; ^3^ Department of Oncology, Jingmen Central Hospital, Jingmen Central Hospital Affiliated to Jingchu University of Technology, Jingmen, Hubei, China

**Keywords:** drug-eluting beads, transarterial chemoembolization, lenvatinib, camrelizumab, unresectable hepatocellular carcinoma, treatment, safety, efficacy

## Abstract

**Objectives:**

The purpose of this study was to compare the safety and efficacy of drug-eluting bead (DEB) transarterial chemoembolization combined with lenvatinib and camrelizumab (DEB-TACE-Len-C) and DEB-TACE-Len for the treatment of unresectable hepatocellular carcinoma (uHCC).

**Methods:**

This retrospective study consecutively included uHCC patients who underwent DEB-TACE-Len-C or DEB-TACE-Len treatment at our hospital and Qujing Second People’s Hospital from April 2020 to April 2022. In total, 85 patients were enrolled. There were 42 patients in the DEB-TACE-Len-C group and 43 patients in the DEB-TACE-Len group. The disease control rate (DCR), objective response rate (ORR), overall survival (OS), progression-free survival (PFS), and adverse events (AEs) were compared between the two groups, and the factors influencing OS and PFS were analysed.

**Results:**

The ORR, DCR, PFS and OS were significantly greater in the DEB-TACE-Len-C group than in the DEB-TACE-Len group (ORR: 76.2% vs. 46.5%, *P* = 0.005; DCR: 88.1% vs. 67.8%, *P* = 0.039; PFS: 10 months vs. 6 months, *P <*0.0001; OS: 24 months vs. 16 months, *P* = 0.0038). Multivariate Cox proportional hazard regression analysis revealed that portal tumour thrombus (PVTT) and therapeutic approach were independent factors affecting PFS and OS. There were no statistically significant differences in the incidence of AEs between the two groups (*P* > 0.05).

**Conclusion:**

Compared with DEB-TACE-Len, DEB-TACE-Len-C is an effective treatment option that can improve the tumour therapeutic response and prolong the OS and PFS in uHCC patients.

## Introduction

Hepatocellular carcinoma (HCC) is the third leading cause of cancer-related mortality worldwide, accounting for approximately 80%-90% of primary liver cancers (PLCs), and its prevalence is predicted to continue to rise (1, 2). However, only a minority of patients can undergo surgery; the majority of patients are diagnosed with advanced HCC (aHCC), for which, non-surgical treatment is the only option (3). Transarterial chemoembolization (TACE) is the most common treatment for patients with unresectable hepatocellular carcinoma (uHCC) and provides local disease control in those with a tolerable liver function or tumour burden (4, 5). Currently, drug-eluting bead TACE (DEB-TACE) has been increasingly employed in HCC treatment. Some studies have shown that, compared with conventional TACE (cTACE), DEB-TACE has the advantages of slower drug release, lower toxicity, and improved efficacy (6, 7). In addition, DEB-TACE also involves fewer treatments than does cTACE (8).

For uHCC patients with Child−Pugh grade A liver function, systemic chemotherapy seems to be the only treatment option. Prior to the advent of molecularly targeted agents (MTAs) with antiangiogenic effects, the survival time for patients with aHCC was limited to 6 months. Systemic therapy for HCC has evolved since the emergence of MTA sorafenib (9). A phase III clinical trial in 2018 revealed that lenvatinib was comparable to sorafenib in terms of overall survival (OS) and that the progression-free survival (PFS) and overall response rate (ORR) of the lenvatinib group were significantly better than those of the sorafenib group (10). The European Association for the Study of the Liver (EASL) and American Association for the Study of Liver Diseases (AASLD) subsequently recommended lenvatinib or sorafenib as the first-line treatment standard for aHCC patients (11, 12). Studies have shown that the ORR and time to progression (TTP) of HCC patients who have undergone DEB-TACE combined with lenvatinib treatment are significantly superior to those of patients who have undergone DEB-TACE combined with sorafenib treatment (13).

Immunotherapy for HCC has been a research hotspot in recent years. Significant progress has been made, which has significantly improved the prognosis in HCC patients. Among these advances, programmed death-1 (PD-1) is a well-studied immunosuppressive molecule. For example, PD-1 inhibitors block the binding of PD-1 to its receptor ligands, thereby activating T lymphocytes and producing long-lasting antitumour effects and inhibiting tumour growth (14). Camrelizumab (Hengrui Medicine Co., Ltd., Jiangsu, China) is a novel PD-1 inhibitor that has been approved by the China Food and Drug Administration (CFADA) for the treatment of HCC (15).

Several studies have demonstrated that TACE combined with targeted drug therapy and immunotherapy can improve the OS of patients with uHCC (16–18). Another study revealed that, compared with cTACE combined with camrelizumab, DEB-TACE combined with camrelizumab for the treatment of uHCC can prolong PFS and the disease control rate (DCR) (19). Therefore, we propose that DEB-TACE combined with camrelizumab and lenvatinib may provide better survival benefits for uHCC patients. This retrospective study was aimed to evaluate the safety and efficacy of DEB-TACE combined with lenvatinib and calceizumab for the treatment of uHCC.

## Materials and methods

### Study design and patient sampling

This was a retrospective study approved by the Ethics Committees of our hospital and Qujing Second People’s Hospital, with no informed consent required. We retrospectively analysed the clinical data collected from uHCC patients who underwent DEB-TACE combined with lenvatinib alone and or with lenvatinib and camrelizumab from April 2020 to April 2022; these data included sex, age, Eastern Cooperative Oncology Group performance status (ECOG PS) score, alpha-fetoprotein (AFP), the absence of laboratory or imaging data such as HBV infection, tumour size, and the number of tumours. A total of 85 patients were included in our study and were divided into three groups: the triple therapy (DEB-TACE-Len-C) group and the dual therapy (DEB-TACE-Len) group. There were 42 patients in the triple therapy group and 43 in the dual therapy group. The inclusion criteria were as follows (1): first diagnosis of HCC according to the European Association for the Study of the Liver (EASL) guidelines (12); (2) age between 18 and 75 years; (3) classification as B or C according to the Barcelona Clinic Liver Cancer (BCLC) system; (4) ECOG PS score of 0 or 1; and (5) Child−Pugh class A or B. The exclusion criteria were as follows: (1) ablation or other treatments prior and during the study period; (2) incomplete clinical data; (3) discontinuation of lenvatinib and camrelizumab due to serious adverse events (AEs); (4) other serious complications, such as heart, lung or renal insufficiency; and (5) Child−Pugh grade C liver function.

### DEB-TACE procedures

All DEB-TACE procedures were performed by interventional radiologists. Hepatic arteriography was performed under local anaesthesia. The right femoral artery was entered via the Seldinger puncture technique, followed by the use of a 5F-arterial access sheath and the introduction of a 5F-Yashiro catheter (Cook, Indiana, USA). Next, 80 mg of epirubicin hydrochloride was loaded into CalliSpheres (Jiangsu Hengrui Pharmaceutical Co., Ltd., Jiangsu, China) of size 100–300 μm or 300–500 μm and subsequently mixed with an iodine contrast agent at a 1:1 ratio. The ratio is made into the final injectable mixture for later use. Afterwards, a 2.6F RAPIDTHRU microcatheter guidewire system (Jiangsu Hengrui Medicine Co., Ltd.) was utilised for superselective tumour catheterization, followed by embolization with a previously prepared mixture under fluoroscopy. As the colour of the polyvinyl alcohol embolization microspheres continued to embolize, the process was terminated once the tumour staining disappeared and blood flow in the tumour-feeding arteries stagnated. Imaging evaluation after DEB-TACE was carried out monthly. DEB-TACE treatment was performed on demand in the same way.

### Lenvatinib treatment

Within 1 or 2 weeks after DEB-TACE treatment, treatment with lenvatinib was initiated if the Child−Pugh score was A or B and if no contraindications to lenvatinib were present. Lenvatinib mesylate capsules (Eisai, Japan) were given once a day at a dose of 8 mg for patients weighing ≤ 60 kg and 12 mg for those weighing >60 kg. If patients experienced grade (PEG) ≥ 3 serious lenvatinib-related adverse events, the dose was adjusted as follows: reduction to 8 mg or 4 mg per day. Dosage reductions or interruptions were permitted if serious adverse events occurred.

### Camrelizumab therapy

Camrelizumab was administered within 2−3 weeks after DEB-TACE treatment. The camrelizumab administration was performed via an intravenous infusion of 200 mg once every 3 weeks. The rate of infusion was reduced or the administration was halted when a low-grade infusion reaction occurred. The dosing was resumed under close observation when the symptoms resolve. Treatment with camrelizumab was continued until unacceptable toxicity occurred. Dose interruptions did not exceed 12 weeks.

All patients included in this study were followed up until October 2022, and all of them returned to the hospital monthly for laboratory tests and imaging examinations. Adverse events (AEs) associated with lenvatinib or camrelizumab were monitored and recorded according to the National Cancer Institute Adverse Event Common Terminology Criteria (Ver. 5.0). The tumour response of all patients, including partial response (PR), complete response (CR), progressive disease (PD) and stable disease (SD), the disease control rate (DCR), and the objective response rate (ORR), was detected according to the modified Response Evaluation Criteria in Solid Tumours (mRECIST). The ORR was defined as the percentage of patients who achieved CR or PR, and the DCR was defined as the percentage of patients who achieved CR, PR or SD. Overall survival (OS) and progression-free survival (PFS) were calculated according to the cut-off dates. PFS was defined as the time interval from the initiation of DEB-TACE treatment until PD, death due to any cause, or the last follow-up. OS was defined as the time from the initiation of DEB-TACE treatment until death or the last follow-up.

### Statistical analysis

All patient data in this study were statistically analysed via SPSS 25.0 and R 4.2.2 software. Categorical variables are presented herein as numbers (percentages), and χ^2^ tests were used for comparisons of categorical variables. Survival curves were plotted via the Kaplan−Meier method and log-rank tests. Cox proportional hazards regression analysis was performed to calculate potential factors that may affect PFS and OS. The proportional hazards assumption was tested with a Cox regression model. Among them, the p value of the BCLC stage and tumour size was less than 0.05, which violates the proportional hazards assumption, and the multivariate results were shown to be insignificant. In the univariate analysis, when *P* was < 0.1, stepwise regression was used to select the model with the lowest AIC for multivariate regression analysis. *P* < 0.05 was considered to indicate statistical significance.

## Results

### Patient baseline data

**Figure 1:**
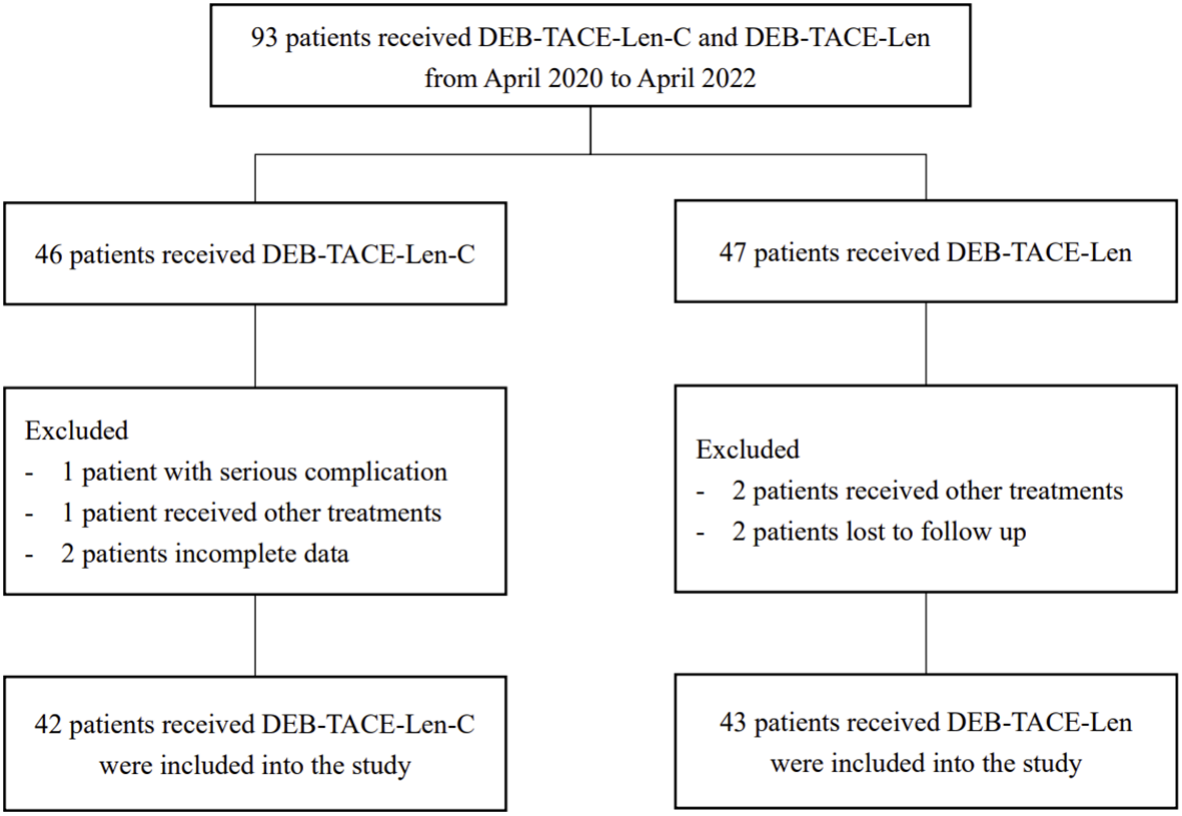
Flow chart of the patient selection: DEB-TACE-Len-C, drug-eluting bead transarterial chemoembolization combined with lenvatinib and camrelizumab; DEB-TACE-Len), drug-eluting bead transarterial chemoembolization combined with lenvatinib.

A total of 93 uHCC patients (53 from Jingmen People’s Hospital, 40 from Qujing Second People’s Hospital) received triple therapy (n = 46) or dual therapy (n = 47) between April 2020 and April 2022. According to the exclusion criteria, 3 patients at Jingmen People’s Hospital and 5 patients at Qujing Second People’s Hospital were excluded. Ultimately, 50 patients at Jingmen People’s Hospital and 35 patients at Qujing Second People’s Hospital were included. Age, sex, BCLC stage, Child−Pugh grade, ECOG PS, liver cirrhosis, aetiology and laboratory parameters were comparable between the two centres, with no significant differences (*P* > 0.05) (**Table 1**). Four patients in the triple therapy group and 4 patients in the dual therapy group were excluded. The flowchart is illustrated in **Figure 1**. Ultimately, 42 patients in the triple therapy group and 43 patients in the dual therapy group were included. The baseline data of the patients were comparable between the groups, with no significant differences (*P* > 0.05) (**Table 2**). The follow-up deadline was October 2022, and the median duration of ow-up was 13.5 months.

**Table 1:** Baseline patient data from the two centres.

Characteristics	Jingmen People’s Hospital	Qujing Second People’s Hospital	*P* value	*P* Adjusted
Age(year)			0.022	0.174
<65	19 (38.0%)	23 (65.7%)		
≥65	31 (62.0%)	12 (34.3%)		
Sex			0.052	0.212
Male	38 (76.0%)	33 (94.3%)		
Female	12 (24.0%)	2 (5.71%)		
ECOG PS score			0.940	1.000
0	13 (26.0%)	8 (22.9%)		
1	37 (74.0%)	27 (77.1%)		
Child−Pugh classification of liver function			0.885	1.000
A	22 (44.0%)	14 (40.0%)		
B	28 (56.0%)	21 (60.0%)		
BCLC stage			0.726	1.000
B	24 (48.0%)	19 (54.3%)		
C	26 (52.0%)	16 (45.7%)		
HBV			0.601	1.000
Yes	41 (82.0%)	31 (88.6%)		
No	9 (18.0%)	4 (11.4%)		
Liver cirrhosis			0.540	1.000
Yes	39 (78.0%)	30 (85.7%)		
No	11 (22.0%)	5 (14.3%)		
PVTT			0.922	1.000
Yes	16 (32.0%)	10 (28.6%)		
No	34 (68.0%)	25 (71.4%)		
Tumour diameter (cm)			0.386	1.000
<10	33 (66.0%)	27 (77.1%)		
≥10	17 (34.0%)	8 (22.9%)		
Number of tumours			1.000	1.000
<3	13 (26.0%)	9 (25.7%)		
≥3	37 (74.0%)	26 (74.3%)		
AFP(ng/ml)			0.597	1.000
<400	34 (68.0%)	21 (60.0%)		
≥400	16 (32.0%)	14 (40.0%)		
Distant organ metastasis			1.000	1.000
Yes	9 (18.0%)	7 (20.0%)		
No	41 (82.0%)	28 (80.0%)		
HAVS			1.000	1.000
Yes	5 (10.0%)	4 (11.4%)		
No	45 (90.0%)	31 (88.6%)		

ECOG, Eastern Cooperative Oncology Group; BCLC Barcelona Liver Cancer Clinic; HBV, hepatitis B virus; AFP, alpha-fetoprotein; PVTT, portal vein tumour thrombus; HAVS, hepatic arterioportal shunt.

**Table 2:** Baseline data of the two groups of patients.

Characteristics	DEB-TACE-Len-C	DEB-TACE-Len	*P* value	*P* Adjusted
Age(year)			0.913	1.000
<65	20 (52.4%)	22 (51.2%)		
≥65	22 (47.6%)	21 (48.8%)		
Sex			0.733	1.000
Male	34 (81.0%)	37 (86.0%)		
Female	8 (19.0%)	6 (14.0%)		
ECOG PS score			0.950	1.000
0	11 (26.2%)	10 (23.3%)		
1	31 (73.8%)	33 (76.7%)		
Child−Pugh classification of liver function			1.000	1.000
A	18 (42.9%)	18 (41.9%)		
B	24 (57.1%)	25 (58.1%)		
BCLC stage			0.233	1.000
B	18 (42.9%)	25 (58.1%0		
C	24 (57.1%)	18 (41.9%)		
HBV			0.963	1.000
Yes	35 (83.3%)	37 (86.0%)		
No	7 (16.7%)	6 (14.0%)		
Liver cirrhosis			1.000	1.000
Yes	34 (81.0%)	35 (81.4%)		
No	8 (19.0%)	8 (18.6%)		
PVTT			0.870	1.000
Yes	12 (28.6%)	14 (32.6%)		
No	30 (71.4%)	29 (67.4%)		
Tumour diameter (cm)			0.684	1.000
<10	31 (73.8%)	39 (67.4%)		
≥10	11 (26.2%)	14 (32.6%)		
Number of tumours			0.420	1.000
<3	13 (31.0%)	9 (20.9%)		
≥3	29 (69.0%)	34 (79.1%)		
AFP(ng/ml)			0.759	1.000
<400	26 (61.9%)	29 (67.4%)		
≥400	16 (38.1%)	14 (32.6%)		
Distant organ metastasis Yes	35 (83.3%)	34 (79.1%)	0.822	1.000
No	7 (16.7%)	9 (20.9%)		
HAVS			0.970	1.000
Yes	37 (88.1%)	39 (90.7%)		
No	5 (11.9%)	4 (9.30%)		

ECOG, Eastern Cooperative Oncology Group; BCLC Barcelona Liver Cancer Clinic; HBV, hepatitis B virus; AFP, alpha-fetoprotein; PVTT, portal vein tumour thrombus; HAVS, hepatic arterioportal shunt.

### Tumour response

The numbers of patients who achieved CR, PR, SD and PD in the triple therapy group were 15 (35.7%), 17 (40.5%), 5 (11.9%), and 5 (11.9%), respectively. The numbers of CR, PR, SD and PD patients in the dual therapy group were 6 (14.0%), 14 (32.6%), 10 (23.3%) and 13 (30.2%), respectively. The ORRs of the triple therapy group and the dual therapy group were 76.2% and 46.5%, the DCRs were 88.1% and 67.8%, respectively, and the differences were statistically significant (the χ^2^ values were 7.880 and 4.276, respectively; *P* values were 0.005 and 0.039, respectively) (**Table 3**).

**Table 3:** Tumour response of the two groups of patients.

Tumour response	DEB-TACE-Len-C	DEB-TACE-Len	*P* value
CR	15(35.7%)	6(14.0%)	
PR	17(40.5%)	14(32.6%)	
SD	5(11.9%)	10(23.3%)	
PD	5(11.9%)	13(30.2%)	
ORR	32(76.2%)	20(46.5%)	0.005
DCR	37(88.1%)	30(67.8%)	0.039

Data are expressed as n (%), CR, complete response; PR, partial response; SD, stable disease; PD, disease progression; ORR, objective response rate; DCR, disease control rate; DEB-TACE-Len-C, drug-eluting bead transarterial chemoembolization combined with lenvatinib and camrelizumab; DEB-TACE-Len, drug-eluting bead transarterial chemoembolization combined with lenvatinib.

### PFS, OS and their influencing factors

**Figure 2:**
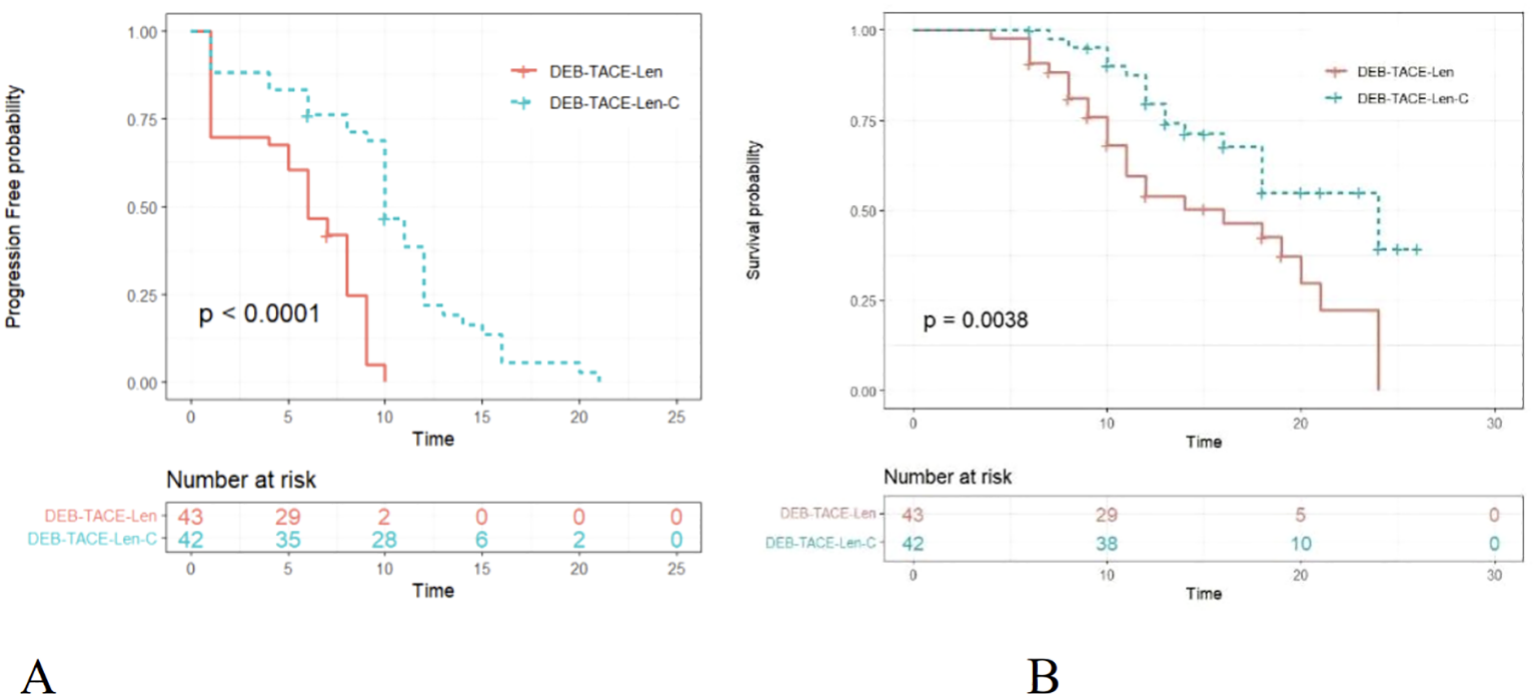
Kaplan–Meier (KM) curves for uHCC patients treated with DEB-TACE-Len-C or DEB-TACE-Len (DEB-TACE-Len-C, drug-eluting bead transarterial chemoembolization combined with lenvatinib and camrelizumab; DEB-TACE-Len, drug-eluting bead transarterial chemoembolization combined with lenvatinib): **(A)** KM curves of the progression-free survival time; **(B)** KM curves of the overall survival time.

By the end of the follow-up, the median PFS times in the triple therapy and dual therapy groups were 10.0 (95% CI: 9.433-10.567) and 6.0 (95% CI: 4.575-7.425) months, respectively, with a statistically significant difference between them (*P <*0.0001; **Figure 2A**). The median OS times in the triple therapy and dual therapy groups, 24 (95% CI: 17.681-30.319) and 16 (95% CI: 9.110-22.890) months, respectively, also significantly differed (*P* = 0.0038; **Figure 2B**).

The results of univariate analysis and multivariate analysis, which may have affected the PFS and OS, are shown in **Tables 4**, **5**. In the univariate analysis, the ECOG PS score (HR:0.565; 95%CI: 0.325-0.981; *P* = 0.040),BCLC stage (HR:2.893; 95%CI: 1.652-5.067; *P* < 0.001), PVTT (HR:7.610; 95%CI: 4.062-14.26; *P* < 0.001), tumour diameter (HR:2.585; 95%CI: 1.487-4.494; *P* < 0.001) distant organ metastasis (HR: 0.200; 95%CI: 0.119-0.336; *P* < 0.001), HAVS (HR:0.118; 95%CI: 0.064~0.219; *P* < 0.001), and treatment method (HR:0.324; 95%CI:0.165~0.636; *P* = 0.001) were the potential factors affecting the PFS, and the potential factors related to OS included BCLC stage (HR:3.070; 95%CI: 1.639~5.752; *P* < 0.001), PVTT (HR:5.347; 95%CI: 2.915~9.807; *P* < 0.001), tumour diameter (HR:3.914; 95%CI: 2.014~7.606; *P* < 0.001), AFP (HR: 2.102; 95%CI: 1.129~3.913; *P* = 0.019), distant organ metastasis (HR: 0.209; 95%CI: 0.112~0.391; *P* < 0.001), HAVS (HR: 0.138; 95%CI: 0.061~0.309; *P* < 0.001) and treatment method (HR: 0.431; 95%CI: 0.234~0.795; *P* = 0.007). In multivariate analysis, two independent factors affecting PFS were identified: PVTT (HR: 8.440; 95%CI: 2.888~22.456; *P* < 0.001) and treatment method (HR:0.203; 95%CI: 0.094~0.440; *P* < 0.001). Two independent factors affected OS: PVTT (HR: 2.834; 95%CI: 1.190~6.750; *P* = 0.019) and treatment method (HR:0.176; 95%CI: 0.082~0.380; *P* < 0.001).

**Table 4:** Prognostic factors for PFS according to univariate and multivariate analyses.

Variable	Univariate analysis		Multivariate analysis	
	HR (95% CI)	*P* value	HR (95% CI)	*P* value
Age (years)		0.897		
<65	Reference			
≥65	1.037(0.602~1.785)			
Sex		0.490		
Male	Reference			
Female	1.293(0.623~2.685)			
ECOG PS score		0.040*		
0	Reference			
1	0.565(0.325~0.981)			
Child−Pugh classification of liver function		0.706		
A	Reference			
B	1.111(0.643~1.917)			
BCLC stage		<0.001***		0.743
B	Reference		Reference	
C	2.893(1.652~5.067)		1.176(0.445~3.101)	
HBV		0.561		
No	Reference			
Yes	0.833(0.450~1.543)			
Liver cirrhosis		0.151		
No	Reference			
Yes	1.859(0.798~4.328)			
PVTT		<0.001***		<0.001***
No	Reference		Reference	
Yes	7.610(4.062~14.26)		8.040(2.888~22.456)	
Tumour diameter (cm)		<0.001***		0.251
<10	Reference		Reference	
≥10	2.585(1.487~4.494)		1.490(0.754~2.946)	
Number of tumours		0.383		
<3	Reference			
≥3	1.331(0.700~2.531)			
AFP(ng/ml)		0.188		0.353
<400	Reference		Reference	
≥400	1.476(0.826~2.637)		1.375(0.702~2.696)	
Distant organ metastasis		<0.001***		0.601
No	Reference		Reference	
Yes	0.200(0.119~0.336)		0.805(0.357~1.814)	
HAVS		<0.001***		
No	Reference			
Yes	0.118(0.064~0.219)			
Treatment		0.001**		<0.001***
DEB-TACE-Len	Reference		Reference	
DEB-TACE-Len-C	0.324(0.165~0.636)		0.203(0.094~0.440)	

HR, hazard ratio; CI, confidence interval; values in bold indicate important information in the statistical analysis section. ECOG, Eastern Cooperative Oncology Group; BCLC Barcelona Liver Cancer Clinic; HBV, hepatitis B virus; AFP, alpha-fetoprotein; PVTT, portal vein tumour thrombus; HAVS, hepatic arteriovenous shunt; DEB-TACE-Len-C, drug-eluting bead transarterial chemoembolization combined with lenvatinib and camrelizumab; DEB-TACE-Len, drug-eluting bead transarterial chemoembolization combined with lenvatinib.

*p<0.05, **p<0.01 ***p<0.001.

**Table 5:** Prognostic factors for OS according to univariate and multivariate analyses.

Variable	Univariate analysis		Multivariate analysis	
	HR (95% CI)	*P* value	HR (95% CI)	*P* value
Age (years)		0.716		
<65	Reference			
≥65	0.896(0.496~1.619)			
Sex		0.321		
Male	Reference			
Female	1.475(0.684~3.183)			
ECOG PS score		0.075		0.940
0	Reference		Reference	
1	0.567(0.304~1.059)		1.030(0.479~2.217)	
Child−Pugh classification of liver function		0.626		
A	Reference			
B	1.162(0.635~2.126)			
BCLC staging		<0.001***		0.114
B	Reference		Reference	
C	3.070(1.639~5.752)		2.200(0.828~5.845)	
HBV		0.904		
No	Reference			
Yes	1.048(0.486~2.263)			
Liver cirrhosis		0.221		
No	Reference			
Yes	1.714(0.723~4.064)			
PVTT		<0.001***		0.019*
No	Reference		Reference	
Yes	5.347(2.915~9.807)		2.834(1.190~6.750)	
Tumour diameter (cm)		<0.001***		0.066
<10	Reference		Reference	
≥10	3.914(2.014~7.606)		2.021(0.954~4.280)	
Number of tumours		0.681		
<3	Reference			
≥3	1.162(0.568~2.377)			
AFP (ng/ml)		0.019*		0.078
<400	Reference		Reference	
≥400	2.102(1.129~3.913)		1.911(0.930~3.924)	
Distant organ metastasis		<0.001***		0.087
No	Reference		Reference	
Yes	0.209(0.112~0.391)		0.501(0.227~1.106)	
HAVS				
No	Reference	<0.001***		
Yes	0.138(0.061~0.309)			
Treatment		0.007*		<0.001***
DEB-TACE-Len	Reference		Reference	
DEB-TACE-Len-C	0.431(0.234~0.795)		0.176(0.082~0.380)	

HR, hazard ratio; CI, confidence interval; values in bold indicate important information in the statistical analysis section. ECOG, Eastern Cooperative Oncology Group; BCLC Barcelona Liver Cancer Clinic; HBV, hepatitis B virus; AFP, alpha-fetoprotein; PVTT, portal vein tumour thrombus; HAVS, hepatic arterioportal shunt; DEB-TACE-Len-C, drug-eluting bead transarterial chemoembolization combined with lenvatinib and camrelizumab; DEB-TACE-Len, drug-eluting bead transarterial chemoembolization combined with lenvatinib.

*p<0.05, ***p<0.001.

### Safety

All patients experienced postembolism syndrome (epigastric abdominal pain, fever, etc.) and/or transient liver function (transaminase) abnormalities after DEB-TACE, but all recovered within a few days. Therefore, the abovementioned common effects after DEB-TACE were not evaluated as AEs in this study. No treatment-associated Grade 4 or 5 adverse events occurred in any patient, so drug treatment was not stopped, but the drug dose was reduced for some patients with grade 3 AEs. A total of 6 patients in the triple therapy group had Grade 3 AEs, including 3 patients (7.1%) with hypertension, 2 patients (4.8%) with nausea and vomiting, and 1 patient (2.4%) with fatigue. Five patients in the dual therapy group experienced Grade 3 AEs, including hypertension in 2 patients (4.7%), nausea and vomiting in 1 patient (2.3%), and fatigue in 2 patients (4.7%). Reactive cutaneous capillary endothelial proliferation (RCCEP) is a unique AE associated with camrelizumab. RCCEP occurred in 7 patients in the triple therapy group, all with Grade 1 or 2 AEs. No statistically significant difference between the groups in terms of the incidence of drug-related adverse effects (AEs) was observed (*P* > 0.05) (**Table 6**).

**Table 6:** Occurrence of AEs in the two groups of patients.

Adverse events	All grades n, (%)		*P* value	Grade 3 n, (%)		*P* value
	DEB-TACE-Len-C (n=42)	DEB-TACE-Len (n=43)		DEB-TACE-Len-C (n=42)	DEB-TACE-Len (n=43)	
Hypertension	12(28.6%)	11(25.6%)	0.756	3(7.1%)	2(4.7%)	0.978
Diarrhoea	6(14.2%)	7(16.3%)	0.799	0(0%)	0(0%)	–
Hand–foot syndrome	9(21.4%)	9(20.9%)	0.955	0(0%)	1(2.3%)	1.000
Nausea and vomiting	13(31.0%)	15(34.9%)	0.700	2(4.8%)	2(4.7%)	1.000
Weakness	10(23.8%)	12(27.9%)	0.666	1(2.4%)	0(0%)	0.991
Loss of appetite	7(16.6%)	8(18.6%)	0.815	0(0%)	0(0%)	–
Hypothyroidism	6(14.3%)	5(11.6%)	0.715	0(0%)	0(0%)	–
Proteinuria	8(19.0%)	7(16.3%)	0.738	0(0%)	0(0%)	–
Bleeding gums	4(9.5%)	4(9.3%)	1.000	0(0%)	0(0%)	–
Gastrointestinal bleeding	2(4.8%)	1(2.3%)	0.983	0(0%)	0(0%)	–
RCCEP	7(16.7%)	0(0%)	0.016	0(0%)	0(0%)	–

RCCEP, reactive cutaneous capillary endothelial proliferation; DEB-TACE-Len-C, drug-eluting bead transarterial chemoembolization combined with lenvatinib and camrelizumab; DEB-TACE-Len, drug-eluting bead transarterial chemoembolization combined with lenvatinib.

## Discussion

TACE is generally considered the first-line treatment for patients with uHCC (16). However, repeated embolization may induce tumour tissue ischaemia and further promote tumour angiogenesis through the upregulation of hypoxia-inducible factor 1-α (13, 14) and angiogenic factors such as VEGF and FGF. In recent years, a small-molecule inhibitor of VEGFR1-3, lenvatinib, has been widely used as a first-line therapy for patients with aHCC and has been demonstrated to be superior to sorafenib (20). Previous studies have explored the use of TACE in combination with molecular targeted drugs for the treatment of uHCC to reduce the frequency of TACE. Numerous studies have demonstrated that TACE or DEB-TACE combined with lenvatinib has favourable therapeutic effects on aHCC patients (21–23). In addition, anti-VEGF therapy enhanced anti-PD-1/anti-PD-L1 efficacy through reversing VEGF-mediated immunosuppression. Combining PD-1 with VEGF inhibitors may improve the immune response of the tumour microenvironment. This combination may increase the infiltration of CD8+ T cells in the TME by temporarily normalising tumour blood vessels, blocking the effects of VEGF, and increasing the value of PD-1 antibodies (24). Therefore, as a PD-1 drug developed in China, camrelizumab, when combined with targeted drugs, can theoretically promote synergistic antitumour effects in TACE. Several studies have confirmed the efficacy of camrelizumab in combination with TACE and targeted drugs in the treatment of uHCC (25, 26). DEB-TACE can be loaded with multiple drugs and enables continuous drug delivery to tumour-feeding arteries while providing permanent embolization, which can maximise the drug efficacy and minimise toxicity (27). Compared with cTACE plus camrelizumab, DEB-TACE plus camrelizumab results in better PFS and tumour response in patients with uHCC (19). Our results revealed that the ORR and DCR in the triple therapy group were significantly greater than those in the dual therapy group. Moreover, the median PFS and OS in the triple therapy group were significantly longer than those in the dual therapy group. The multivariate analysis results suggested that combination therapy with camrelizumab was a favourable independent predictor of OS and PFS, whereas a portal vein tumour thrombus (PVTT) was an independent risk factor for PFS and OS. PVTT occurs in approximately 44%-62% of HCC patients and plays an essential role in prognosis and clinical staging, and HCC patients with PVTT usually have a poorer prognosis (28). In the present study, a Cox model was implemented to reduce potential factors that might affect the results. Compared with patients without PVTT, patients with PVTT had an increased risk of tumour progression. From this perspective, our data were consistent with those of previous studies.

Common AEs in both groups, including hypertension and hand−foot syndrome, affect the long-term quality of life of patients to a certain extent, but these complications are not fatal. RCCEP is an AE related only to camrelizumab. Liang Yin et al. confirmed that RCCEP is not only an AE of camrelizumab treatment of HCC but also an important predictor of better prognosis of TACE and camrelizumab in the treatment of advanced HCC (14). In this study, the AEs of patients in the two groups were controllable, and no Grade 4 or 5 AEs occurred. Moreover, there were no statistically significant differences between the two groups in terms of any AEs except for RCCEP. These results indicate that the camrelizumab combination does not significantly increase the risk of AEs compared with DEB-TACE combined with lenvatinib.

However, our study has several limitations. First, this investigation was retrospective and may have had selection bias. Second, the sample size of patients was small, only two centres were included; therefore, the nature of the patients may limit their representativeness. Since our sample size is relatively small, we may have generated a Type II error; further studies with larger sample sizes are needed to reduce the risk of making such errors. In addition, our follow-up time was insufficient. Future multicentre prospective clinical studies with larger sample sizes and longer follow-up periods are needed to further confirm the efficacy of DEB-TACE used in combination with lenvatinib and camrelizumab.

## Conclusion

In conclusion, the triple therapy with DEB-TACE in combination with lenvatinib and camrelizumab is a reliable and effective therapeutic choice for uHCC patients. This combination treatment significantly improves the tumour response, prolongs patient survival, and has tolerable adverse effects.

## Data Availability

The raw data supporting the conclusions of this article will be made available by the authors, without undue reservation.

## References

[1] Kudo M Ueshima K Chan S Minami T Chishina H Aoki T. Lenvatinib as an initial treatment in patients with intermediate-stage hepatocellular carcinoma beyond up-to-seven criteria and child-pugh A liver function: A proof-of-concept study. Cancers (Basel). (2019) 11:1084. doi: 10.3390/cancers11081084 31370183 PMC6721438

[2] Chen S Wu Z Shi F Mai Q Wang L Wang F. Lenvatinib plus TACE with or without pembrolizumab for the treatment of initially unresectable hepatocellular carcinoma harbouring PD-L1 expression: a retrospective study. J Cancer Res Clin Oncol. (2022) 148:2115–25. doi: 10.1007/s00432-021-03767-4 PMC9293824 34453221

[3] Chen R Li L Li Y Song K Shen C Ma P. Efficacy and safety of transcatheter arterial chemoembolization-lenvatinib sequential therapy for patients with unresectable hepatocellular carcinoma: a single-arm clinical study. J Gastrointest Oncol. (2022) 13:1367–75. doi: 10.21037/jgo-22-525 PMC9274079 35837188

[4] Cai M Huang W Huang J Shi W Guo Y Liang L. Transarterial chemoembolization combined with lenvatinib plus PD-1 inhibitor for advanced hepatocellular carcinoma: A retrospective cohort study. Front Immunol. (2022) 13:848387. doi: 10.3389/fimmu.2022.848387 35300325 PMC8921060

[5] Chai B Xiang D Wang W Ren Y Wang F Wang J. Arterial enhancement fraction in evaluating the therapeutic effect and survival for hepatocellular carcinoma patients treated with DEB-TACE. Cancer Imaging. (2022) 22:38. doi: 10.1186/s40644-022-00477-z 35908071 PMC9338623

[6] Ji K Zhu H Wu W Li X Zhan P Shi Y. Tumor response and nomogram-based prognostic stratification for hepatocellular carcinoma after drug-eluting beads transarterial chemoembolization. J Hepatocell Carcinoma. (2022) 9:537–51. doi: 10.2147/JHC.S360421 PMC9188409 35698645

[7] Shi Q Chen D Zhou C Liu J Huang S Yang C. Drug-eluting beads versus lipiodol transarterial chemoembolization for the treatment of hypovascular hepatocellular carcinoma: A single-center retrospective study. Cancer Manag Res. (2020) 12:5461–8. doi: 10.2147/CMAR.S255960 PMC7351634 32753963

[8] Kloeckner R Weinmann A Prinz F Pinto dos Santos D Ruckes C Dueber C. Conventional transarterial chemoembolization versus drug-eluting bead transarterial chemoembolization for the treatment of hepatocellular carcinoma. BMC Cancer. (2015) 15:465. doi: 10.1186/s12885-015-1480-x 26059447 PMC4460638

[9] Sho T Morikawa K Kubo A Tokuchi Y Kitagataya T Yamada R. Prospect of lenvatinib for unresectable hepatocellular carcinoma in the new era of systemic chemotherapy. World J Gastrointest Oncol. (2021) 13:2076–87. doi: 10.4251/wjgo.v13.i12.2076 PMC8713309 35070043

[10] Kudo M Finn RS Qin S Han KH Ikeda K Piscaglia F. Lenvatinib versus sorafenib in first-line treatment of patients with unresectable hepatocellular carcinoma: a randomised phase 3 non-inferiority trial. Lancet. (2018) 391:1163–73. doi: 10.1016/S0140-6736(18)30207-1 29433850

[11] Marrero JA Kulik LM Sirlin CB Zhu AX Finn RS Abecassis MM. Diagnosis, staging, and management of hepatocellular carcinoma: 2018 practice guidance by the american association for the study of liver diseases. Hepatology. (2018) 68:723–50. doi: 10.1002/hep.29913 29624699

[12] European Association for the Study of the Liver. Electronic address: easloffice@easloffice.eu; European Association for the Study of the Liver. EASL Clinical Practice Guidelines: Management of hepatocellular carcinoma. J Hepatol. (2018) 69:182–236. doi: 10.1016/j.jhep.2018.03.019 29628281

[13] Xue M Wu Y Zhu B Zou X Fan W Li J. Advanced hepatocellular carcinoma treated by transcatheter arterial chemoembolization with drug-eluting beads plus lenvatinib versus sorafenib, a propensity score matching retrospective study. Am J Cancer Res. (2021) 11:6107–18. PMC8727795 35018245

[14] Yin L Liu KC Lv WF Xu SB Lu D Zhou CZ. Predicting outcome in combination treatment of TACE and camrelizumab for advanced hepatocellular carcinoma: tumor hypervascularity and reactive cutaneous capillary endothelial proliferation. Drug Des Devel Ther. (2022) :16:3421–3429. doi: 10.2147/DDDT.S372276 PMC9531610 36203820

[15] Xia Y Tang W Qian X Li X Cheng F Wang K. Efficacy and safety of camrelizumab plus apatinib during the perioperative period in resectable hepatocellular carcinoma: a single-arm, open label, phase II clinical trial. J Immunother Cancer. (2022) 10:e004656. doi: 10.1136/jitc-2022-004656 35379737 PMC8981365

[16] Liu J Li Z Zhang W Lu H Sun Z Wang G. Comprehensive treatment of trans-arterial chemoembolization plus lenvatinib followed by camrelizumab for advanced hepatocellular carcinoma patients. Front Pharmacol. (2021) 12:709060. doi: 10.3389/fphar.2021.709060 34733154 PMC8558352

[17] Cao F Yang Y Si T Luo J Zeng H Zhang Z. The efficacy of TACE combined with lenvatinib plus sintilimab in unresectable hepatocellular carcinoma: A multicenter retrospective study. Front Oncol. (2021) 11:783480. doi: 10.3389/fonc.2021.783480 34988019 PMC8721033

[18] Sun B Zhang L Sun T Ren Y Cao Y Zhang W. Safety and efficacy of lenvatinib combined with camrelizumab plus transcatheter arterial chemoembolization for unresectable hepatocellular carcinoma: A two-center retrospective study. Front Oncol. (2022) :982948. doi: 10.3389/fonc.2022.982948 36172158 PMC9511022

[19] Ren Y Guo Y Chen L Sun T Zhang W Sun B. Efficacy of drug-eluting beads transarterial chemoembolization plus camrelizumab compared with conventional transarterial chemoembolization plus camrelizumab for unresectable hepatocellular carcinoma. Cancer Control. (2022) 29:10732748221076806. doi: 10.1177/10732748221076806 35343254 PMC8958708

[20] Al-Salama ZT Syed YY Scott LJ. Lenvatinib: A review in hepatocellular carcinoma. Drugs. (2019) 79:665–74. doi: 10.1007/s40265-019-01116-x 30993651

[21] Peng Z Fan W Zhu B Wang G Sun J Xiao C. Lenvatinib combined with transarterial chemoembolization as first-line treatment for advanced hepatocellular carcinoma: A phase III, randomized clinical trial (LAUNCH). J Clin Oncol. (2023) 41:117–27. doi: 10.1200/JCO.22.00392 35921605

[22] Xia D Bai W Wang E Li J Chen X Wang Z. Lenvatinib with or without concurrent drug-eluting beads transarterial chemoembolization in patients with unresectable, advanced hepatocellular carcinoma: A real-world, multicenter, retrospective study. Liver Cancer. (2022) 11:368–82. doi: 10.1159/000523849 PMC9294948 35978600

[23] Kawamura Y Kobayashi M Shindoh J Kobayashi Y Okubo S Tominaga L. Lenvatinib-Transarterial Chemoembolization Sequential Therapy as an Effective Treatment at Progression during Lenvatinib Therapy for Advanced Hepatocellular Carcinoma. Liver Cancer. (2020) 9:756–70. doi: 10.1159/000510299 PMC7768146 33442544

[24] Shigeta K Matsui A Kikuchi H Klein S Mamessier E Chen IX. Regorafenib combined with PD1 blockade increases CD8 T-cell infiltration by inducing CXCL10 expression in hepatocellular carcinoma. J Immunother Cancer. (2020) 8:e001435. doi: 10.1136/jitc-2020-001435 33234602 PMC7689089

[25] Zhang JX Chen YX Zhou CG Liu J Liu S Shi HB. Efficacy and safety of the combination of transarterial chemoembolization with camrelizumab plus apatinib for advanced hepatocellular carcinoma: A retrospective study of 38 patients from a single center. Can J Gastroenterol Hepatol. (2022) 2022:7982118. doi: 10.1155/2022/7982118 35586608 PMC9110252

[26] Ju S Zhou C Yang C Wang C Liu J Wang Y. Apatinib plus camrelizumab with/without chemoembolization for hepatocellular carcinoma: A real-world experience of a single center. Front Oncol. (2022) 11:835889. doi: 10.3389/fonc.2021.835889 35174073 PMC8841670

[27] Nouri YM Kim JH Yoon HK Ko HK Shin JH Gwon DI. Update on transarterial chemoembolization with drug-eluting microspheres for hepatocellular carcinoma. Korean J Radiol. (2019) 20:34–49. doi: 10.3348/kjr.2018.0088 30627020 PMC6315076

[28] Ding X Sun W Li W Shen Y Guo X Teng Y. Transarterial chemoembolization plus lenvatinib versus transarterial chemoembolization plus sorafenib as first-line treatment for hepatocellular carcinoma with portal vein tumor thrombus: A prospective randomized study. Cancer. (2021) 127:3782–93. doi: 10.1002/cncr.33677 34237154

